# One-Pot Microfluidics to Engineer Chitosan Nanoparticles Conjugated with Antimicrobial Peptides Using “Photoclick” Chemistry: Validation Using the Gastric Bacterium *Helicobacter pylori*

**DOI:** 10.1021/acsami.3c18772

**Published:** 2024-03-14

**Authors:** Diana R. Fonseca, Pedro M. Alves, Estrela Neto, Beatriz Custódio, Sofia Guimarães, Duarte Moura, Francesca Annis, Marco Martins, Ana Gomes, Cátia Teixeira, Paula Gomes, Rúben F. Pereira, Paulo Freitas, Paula Parreira, M. Cristina L. Martins

**Affiliations:** † i3S − Instituto de Investigação e Inovação em Saúde, Universidade do Porto, Rua Alfredo Allen, 208, 4200-135 Porto, Portugal; ‡ Instituto Nacional de Engenharia Biomédica, Universidade do Porto, R. Alfredo Allen 208, 4200-135 Porto, Portugal; § Faculdade de Engenharia, Departamento de Engenharia Metalúrgica e de Materiais, Universidade do Porto, R. Dr. Roberto Frias, 4200-465 Porto, Portugal; ∥ LAQV-REQUIMTE, Departamento de Química e Bioquímica, Faculdade de Ciências, Universidade do Porto, Rua do Campo Alegre 685, 4169-007 Porto, Portugal; ⊥ ICBAS−Instituto de Ciências Biomédicas Abel Salazar, Universidade do Porto, Rua de Jorge Viterbo Ferreira, 4050-313 Porto, Portugal; # INL, International Iberian Nanotechnology Laboratory, Av. Mte. José Veiga s/n, 4715-330 Braga, Portugal; ¶ INESC-MN, INESC Microsystems and Nanotechnologies, Rua Alves Redol 9, 1000-029 Lisboa, Portugal

**Keywords:** biomaterials, covalent immobilization, Helicobacter pylori, microfluidic systems, MSI-78A, norbornene, surface modification, Thiol−ene click chemistry, microfluidics

## Abstract

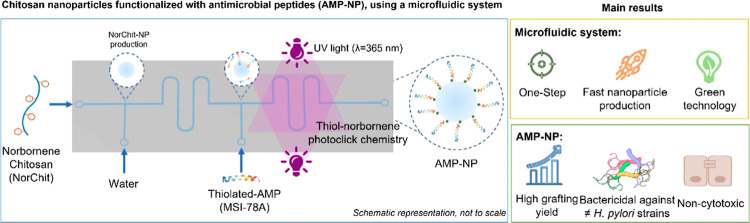

Surface bioconjugation of antimicrobial peptides (AMP) onto nanoparticles (AMP-NP) is a complex, multistep, and time-consuming task. Herein, a microfluidic system for the one-pot production of AMP-NP was developed. Norbornene-modified chitosan was used for NP production (NorChit-NP), and thiolated-AMP was grafted on their surface via thiol–norbornene “photoclick” chemistry over exposure of two parallel UV LEDs. The MSI-78A was the AMP selected due to its high activity against a high priority (level 2) antibiotic-resistant gastric pathogen: *Helicobacter pylori (H. pylori)*. AMP-NP (113 ± 43 nm; zeta potential 14.3 ± 7 mV) were stable in gastric settings without a cross-linker (up to 5 days in pH 1.2) and bactericidal against two highly pathogenic *H. pylori* strains (10^11^ NP/mL with 96 μg/mL MSI-78A). Eradication was faster for *H. pylori* 26695 (30 min) than for *H. pylori* J99 (24 h), which was explained by the lower minimum bactericidal concentration of soluble MSI-78A for *H. pylori* 26695 (32 μg/mL) than for *H. pylori* J99 (128 μg/mL). AMP-NP was bactericidal by inducing *H. pylori* cell membrane alterations, intracellular reorganization, generation of extracellular vesicles, and leakage of cytoplasmic contents (transmission electron microscopy). Moreover, NP were not cytotoxic against two gastric cell lines (AGS and MKN74, ATCC) at bactericidal concentrations. Overall, the designed microfluidic setup is a greener, simpler, and faster approach than the conventional methods to obtain AMP-NP. This technology can be further explored for the bioconjugation of other thiolated-compounds.

## 1 Introduction

Antibiotic resistance is growing at a higher speed than expected, being considered a public health issue as well as a threat to global health and development.^1^*Helicobacter pylori (H. pylori)*, a Gram-negative bacterium that infects the gastric mucosa of 50% of the world population, was considered by the World Health Organization (WHO) as one of the antibiotic-resistant bacteria for which it is urgent to develop alternative treatments (the failure of current therapies is estimated at 10–40%).^2−4^ Therefore, antibiotic-free bioengineered strategies against this pathogen, even when organized in biofilms, have gained relevance.^5−7^ Chitosan nano/microparticles have been developed (i) as gastric drug delivery systems to protect drugs from the harsh gastric environment (e.g., mannose,^8^ rhamnolipids,^9^ curcumin,^10^ and antimicrobial peptides—AMP^11^), (ii) to kill,^12^ or (iii) to bind and remove *H. pylori* from the stomach.^13^ Although few AMPs (MSI-78 and its analogue MSI-78A)^14,15^ are effective against *H. pylori*, they are a promising therapeutic strategy to counteract gastric infection due to their bactericidal effect coupled with a low propensity for resistance development.^11^ The encapsulation of MSI-78 into chitosan-alginate nanoparticles (NP) increases the effectiveness of the clearance of *H. pylori* infection in a mice model when compared with MSI-78 administered in suspension. Also, this nanosystem did not induce resistance after 12 passages (*in vitro*), contrarily to what occurred with the commonly prescribed antibiotics to overcome *H. pylori* infection (metronidazole, clarithromycin, and amoxicillin).^11^ Nonetheless, this strategy did not protect AMP from self-aggregation or proteolytic degradation after *in vivo* delivery, hampering its potential as a new therapeutic approach.^16−18^ To overcome these setbacks, MSI-78A was previously grafted onto chitosan microspheres (AMP-ChMic, ∼4 μm diameter), which improved *H. pylori* bactericidal activity in comparison to free-MSI-78A.^19^ However, better performance could be achieved when reducing the particle size from micro to nano due to (i) the higher area/volume ratio to bind AMP; (ii) the ability to contact/penetrate and kill *H. pylori*; and (iii) the capacity to cross the gastric mucus barrier (reaching the infection local). Nevertheless, the production of AMP-NP using the conventional protocols is a time-consuming multistep process (e.g., spray drying, genipin cross-linking, PEG-spacer conjugation and, finally, AMP conjugation).^19,20^

**Figure 1:**
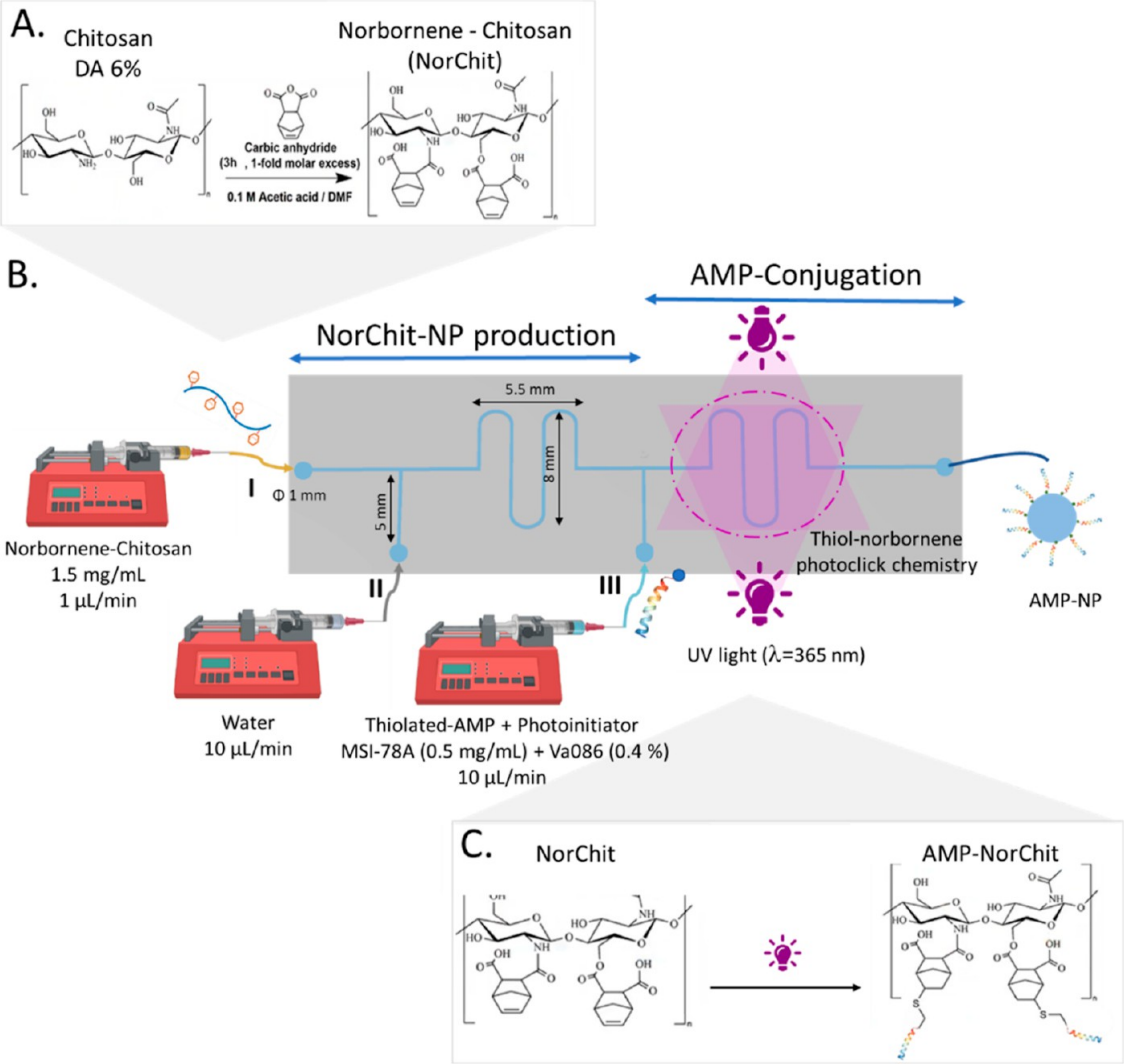
Norbornene-chitosan NP production and thiolated-AMP grafting. (A) Norbornene groups were conjugated onto chitosan upon reaction with carbic anhydride in an acetic acid/*N*,*N*-dimethylformamide (DMF) cosolvent system to obtain NorChit, as described by us.^21^ (B) Microfluidics setup: the main channel is 36.5 mm long, and all channels are 200 μm wide and 80 μm deep. Optimized AMP-NP production: NorChit solution (1.5 mg/mL, 1 μL/min) was added to the main channel, with the NorChit-NP being formed in the intersection with type I water (10 μL/min). NorChit-NP stabilization occurred in the first serpentine, followed by the introduction of AMP (0.5 mg/mL; 10 μL/min) with the photoinitiator (VA-086, 0.4% w/v) in the system. Solutions were introduced in the system using Tygon ND 100–80 tubing (0.76 × 2.29 mm) coupled with stainless steel straight (20G), both from darwin microfluidics. (C) Thiol-norbornene photoclick chemistry (TNPC) was triggered by two UV lights (λ = 365 nm; superior LED: 35 mW/cm^2^; inferior LED: 75 mW/cm^2^) in parallel, followed by final AMP-NP collection. Schematic representation, not to scale; partially created with BioRender.com.

In this work, we intended to develop a straightforward microfluidic system to prepare chitosan NP functionalized with MSI-78A (AMP-NP) in a single step. Several optimizations were performed to adjust NP size, concentration, peptide grafting, and, consequently, overall NP bactericidal activity, namely, by adjusting the norbornene-chitosan and AMP concentrations, as well as UV light intensities (based on the number and position of UV LED). The optimized “one-pot” microfluidic device (Figure 1) uses chitosan functionalized with norbornene groups^21^ to produce NP (NorChit-NP) at the intersection with the water inlet. A thiolated AMP (MSI-78A-SH) is injected in the third inlet to be grafted onto NorChit-NP (AMP-NP) inside the microfluidics via thiol–norbornene “photoclick” chemistry (TNPC) upon exposure to two parallel UV LEDs in the system.

AMP-NP were characterized regarding their size, shape, stability in gastric-like conditions, and concentration by NP tracking analysis (NTA). NP zeta potential was assessed by dynamic light scattering (DLS), the success of the AMP graft by FTIR and confocal Raman microspectroscopy, and their round-morphology by transmission electron microscopy (TEM). The amount of grafted AMP was assessed by amino acid analysis (AAA). AMP-NP *in vitro* efficacy was evaluated against two highly pathogenic *H. pylori* strains (*H. pylori* J99 and *H. pylori* 26695), and their cytotoxicity profile was evaluated using two gastric cell lines (AGS and MKN74, ATCC).

## 2 Materials and Methods

### 2.1 Development of Antimicrobial Peptide-Grafted Nanoparticles

AMP (MSI-78A-SH: GIGKFLKKAKKFAKAFVKILKK-Ahx-C) was synthesized with a flexible 6-aminohexanoic acid (Ahx, Novabiochem) spacer and a cysteine (Novabiochem) at the C-terminus, as previously described.^14,19^

Norbornene-modified chitosan (NorChit; Figure 1A) was prepared by us according to Alves *et al.*(21) Briefly, purified squid chitosan (France Chitine) with a degree of acetylation (DA) of 2–3% (^1^H NMR and FTIR chitosan spectra and respective DA calculations are in S1 and S2) and molecular weight of 363 ± 28 kDa was solubilized in 0.1 M acetic acid (AppliChem) with a final concentration of 0.4% w/v. To introduce the norbornene groups, chitosan in solution reacted with carbic anhydride in *N*,*N*-dimethylformamide (DMF; 0.15 M, Merck), which was added every 30 min, three times. The reaction proceeded for 3 h at room temperature (RT). The mix was then dialyzed (10 kDa cutoff; Thermo Scientific SnakeSkin) against decreasing concentrations of sodium chloride (NaCl; VWR chemicals) in type II water (purified water with a resistivity of >1 MΩ cm, a conductivity of <1 μS/cm, and <50 ppb of total organic carbons) for 4 days. The dialysis buffer was changed thrice every day and kept at 40 °C during the first 48 h. The resultant NorChit was frozen, lyophilized (−50 °C), and stored at −20 °C, under an inert (nitrogen) atmosphere until further use.

NorChit-NP was generated in the microfluidic system (Figure 1B). The microfluidic design and flow rates were first screened and adjusted based on the literature.^22−27^ Three different concentrations of NorChit solution (1.5, 2, and 2.5 mg/mL) were tested, and the final selection was based on the size and concentration of the NorChit-NP obtained. To evaluate the need for cross-linking, NorChit-NP was prepared with and without 17.5 mg/mL of dithiothreitol (DTT, ZYTech), and their quantification was done by NTA before and after immersion in simulated gastric fluid (SGF; pH 1.2, composed of 0.2 M hydrochloric acid and 0.2 M sodium chloride, both from VWR) for 3 h (to simulate the digestion time) and 120 h (stability for storage) at 150 rotations per minute (rpm) and 37 °C.

#### 2.1.1 Microfluidic System

The microfluidic chip (Figure 1B) was designed using OpenSCAD software (version 2019.05). The main channel is 36.5 mm long, and all channels are 200 μm wide and 80 μm depth. All of the other dimensions are detailed in Figure 1B.

For printing, microfluidic design was checked for printability, and scaffolds were added accordingly using PreForm software (Preform 3.0.1, Formlabs). The microfluidic molds were then 3D printed in a stereolithography Form 3 printer (Formlabs; resolution 25 μm) with a Clear Resin (Clear V4 resin; Formlabs). These molds were postprocessed by two rounds of immersion in isopropanol (Enzymatic, S.A.) for 15 min to remove uncured resin, followed by air-drying and an overnight heat treatment at 80 °C. Poly dimethylsiloxane (PDMS, Sylgard 184, Dow Corning) was then cast into the mold with a 10:1 (w/w) ratio of base and curing agent and thermally cured for 2 h at 70 °C. Then, each PDMS slab was separated from the mold, the inlets and the outlet were punched out with a 1 mm biopsy punch (KAI medical, BP-50F) and then cleaned with residue-free tape (Tape 3 M 471 50 mm × 33 m; Rodrigues & Boaventura, LDA). The PDMS layer was mounted on top of a clean and treated (Alconox (1% v/v)) glass coverslips, slightly pressing both surfaces after an oxygen plasma treatment [Tergeo Plasma Cleaner, Pie Scientific (TG100)].

#### 2.1.2 AMP-NP Production in the Microfluidic System

The inlet solutions were prepared immediately before use. NorChit solution (1.5 mg/mL) was prepared by NorChit hydration in type I water (ultrapure water with a resistivity >18 MΩ cm, a conductivity of <0.056 μS/cm, and <50 ppb of total organic carbons; Merck Millipore) for 8 h under slow stirring at 4 °C. Then, glacial acetic acid (0.1 M; AppliChem Panreac) was added and left overnight under moderate stirring at RT. AMP solution (0.5 mg/mL) was prepared by MSI-78A-SH resuspension in phosphate buffer (pH 6.6). The photoinitiator VA-086 (2,2′-azobis[2-methyl-*N*-(2-hydroxyethyl)propionamide]; Wako Chemicals) was included in the AMP solution at 0.4% (w/v).

NorChit solution was injected (1 μL/min) as the main flow [Figure 1B(I)], and type I water was injected (10 μL/min) in the first lateral channel [Figure 1B(II)] of the microfluidic system using syringe pumps (New era)—Figure 1B. The NorChit-NP was formed in the intersection of both solutions. Simultaneously, AMP (MSI-78A-SH) solution was injected (10 μL/min) into the second lateral channel [Figure 1B(III)], and the AMP was grafted onto the NorChit-NP surface by TNPC^21,28^ (Figure 1C) in the presence of two parallel UV-LED sources (λ = 365 nm, Mouser Electronics Inc.; distance to the microfluidic system: top LED ∼4 cm, bottom LED ∼0.5 cm; intensity: top LED 35 mW/cm^2^, bottom LED 75 mW/cm^2^; superimposed light intensity received by the NP 110 mW/cm^2^) in the microfluidic system (Figure 1B). AMP-NP was collected, filtered with an Amicon filtration system (50 kDa; Merck), and rinsed with type I water by centrifugation at 816*g* for 5 min (Eppendorf 5417R) to remove unbound AMP. The obtained pellet was resuspended in type I water.

The same procedure was carried out (i) without AMP (NorChit-NP; NP control) and (ii) using non-thiolated MSI-78A to quantify the maximum AMP adsorption onto NorChit-NP (covalent grafting will not occur without the –SH group).

Prior to NP preparation, the workbench was thoroughly cleaned with ethanol (70% v/v; VWR), and NP was collected in sterile microcentrifuge tubes (covered with parafilm). A control of sterilization was performed for all assays by inoculating 20 μL of NP solution onto tryptic soy agar (TSA; Sigma-Aldrich) plates. Incubation proceeded for 24 h at 37 °C.

##### 2.1.2.1 UV Light Intensity Optimization

To establish the required ultraviolet (UV) light (λ = 365 nm) intensity (1 LED, 2 LED in parallel, and 3 LED in series) for peptide grafting using TNPC (Figure S3—Supporting Information), a model peptide (CGGGGRGDSP) with a fluorescein isothiocyanate (FITC) tag (0.25 and 0.5 mg/mL) was used.

##### 2.1.2.2 AMP Concentration Optimization

AMP (MSI-78A-SH) concentration (0.25; 0.5; and 1 mg/mL) was selected based on the bactericidal effect of the AMP-NP produced (Figure S4—Supporting Information).

#### 2.1.3 Nanoparticle Characterization

##### 2.1.3.1 Nanoparticle Tracking Analysis

NP concentrations were determined using a NanoSight NS300 NTA Dev Build 3.2.16 instrument equipped with an sCMOS camera (Malvern Instruments). Samples were diluted (1:100 to 1:1000 in type I water) to obtain a concentration within a scale of magnitude of 10^8^ NP/mL. Three movies of 30 s were recorded for each sample (with a threshold of 10 to 50 particles per frame). Data acquisition and processing were performed using NanoSight NS300 NTA 3.0 software.

##### 2.1.3.2 Dynamic Light Scattering

Zeta potential measurements were made in a Horiba SZ-100Z DLS system (Horiba Scientific) at the International Iberian Nanotechnology Laboratory facilities. The samples (NorChit-NP and AMP-NP) were diluted 1:200 in 10 mM sodium chloride (NaCl). Measurements were taken at 25 °C, with 3 measurements per each sample and automatic voltage selection.

##### 2.1.3.3 Fourier-Transform Infrared Spectroscopy

The AMP grafting onto NorChit-NP was assessed by attenuated total reflectance FTIR (ATR-FTIR; Universal ATR module coupled to a PerkinElmer Frontier) at the i3S—*Instituto de Investigação e Inovação em Saúde*—Biointerfaces and Nanotechnology scientific platform. Samples were directly placed in the cell and left to dry at RT for 10 min. Spectra were obtained using 32 scans at 4 cm^–1^ spectral resolution, with the wavenumber ranging from 3500 to 750 cm^–1^.

##### 2.1.3.4 Confocal Raman Microspectroscopy

AMP grafting onto NorChit-NP was also confirmed by confocal Raman microspectroscopy (Confocal Raman/FTIR Microscope, LabRAM HR800 UV, Horiba Jobin-Yvon), with a 515 nm diode laser at the i3S Bioimaging Scientific Platform.

Spectra were acquired using a 100*x* MPlan 0.99 NA objective within the fingerprint range of 750–3500 cm^–1^ with a spectral resolution of 2.14 cm^–1^ with an acquisition time of 15 s and two accumulations. The pinhole was set to 100 μm. Measurements were made at 3 random areas of lyophilized samples (overnight, −50 °C).

##### 2.1.3.5 Transmission Electron Microscopy

The morphology of the NP was evaluated through negative staining with TEM. 10 μL of each sample was mounted on Formvar/carbon film-coated mesh nickel grids (Electron Microscopy Sciences, Hatfield, PA). After 2 min, the liquid in excess was removed with filter paper, 10 μL of 1% uranyl acetate was added to the grids and incubated for 10 s, followed by the removal of the liquid in excess with filter paper. Samples were visualized on a JEOL JEM 1400 TEM at 120 kV (Tokyo) at i3S Histology and Electron Microscopy Service scientific platform.

##### 2.1.3.6 Amino Acid Analysis

The concentration of AMP grafted onto NP was also directly determined using reverse-phase chromatography after hydrolysis.^29^ First, samples were hydrolyzed using 6 M aqueous hydrochloric acid (Fisher Chemical) containing 10% v/v phenol (Sigma-Aldrich) at 110 °C for 24 h. Then, the samples were dried (evaporation of the solvent) and dissolved in high-performance liquid chromatography (HPLC)-grade water with aminobutyric acid as an internal standard. The AccQ-Tag protocol from Waters was performed for derivatization of the amino acids released, allowing their quantification by analytical HPLC (Waters 600) with a Waters 2487 UV-detector (λ = 254 nm).^30^ AMP adsorbed onto NP was also quantified, and NorChit-NP was used as a control.

### 2.2 In Vitro Efficacy Assays

The antimicrobial efficacy of AMP-NP and NorChit-NP against *H. pylori* was evaluated in vitro using two highly pathogenic (cytotoxin associated gene A (CagA) and vacuolating cytotoxin A (VacA) positive) clinical isolates: *H. pylori* J99 (ATCC 700824), isolated from a patient with a duodenal ulcer,^31^ and *H. pylori* 26695 (ATCC 700392), isolated from a patient suffering from gastritis.^32^

#### 2.2.1 *Helicobacter pylori* Growth

*H. pylori* was routinely grown in Blood Agar solid plates (BA; Oxoid) supplemented with 10% (v/v) of defibrinated horse blood (Probiologica) and 0.2% (v/v) of an antibiotic-cocktail (0.155 g/L polymyxin B, 6.25 g/L vancomycin, 1.25 g/L amphotericin B, and 3.125 g/L trimethoprim; all from Sigma-Aldrich), for 48 h at 37 °C under a microaerophilic environment (GenBox System, BioMérieux) as described.^14^ Then, some colonies were streaked onto a fresh BA plate and incubated under the same conditions. After 48 h, bacteria were transferred to Brucella Broth medium (BB, Oxoid) supplemented with 10% of fetal bovine serum (FBS, Gibco) with optical density (OD) adjusted to 0.1 (λ = 600 nm; UV/vis spectrophotometer, Lambda 45, PerkinElmer), and grown overnight under microaerophilic and dynamic (150 rpm) conditions at 37 °C. For all the assays, the bacterial inoculum was adjusted to 0.03 OD at 600 nm (OD_600_), which corresponds to approximately 1 × 10^7^ colony-forming units (cfus)/mL.^19^

#### 2.2.2 AMP-NP Bactericidal Activity

The ability of AMP-NP to impair *H. pylori* J99 and *H. pylori* 26695 growth was assessed by performing a time-to-kill kinetic assay. Before the assays, AMP-NP and NorChit-NP were centrifuged (816 *g*, 10 min, Amicon 50 kDa) and suspended in supplemented liquid media to achieve a final concentration of 10^11^ NP/mL, the maximum concentration obtained in each microfluidic batch.

*H. pylori* was incubated with AMP-NP and NorChit-NP at 10^11^ NP/mL, 37 °C, and 150 rpm under microaerophilic conditions. After 30 min, 1, 2, 4, 6, and 24 h, samples were collected, serially diluted in sterile phosphate-buffered saline (PBS; 0.1 M; pH 7.4), and plated in fresh BA plates. Pure cultures of *H. pylori* and AMP in solution at the previously determined minimum bactericidal concentration (MBC) (32 μg/mL for *H. pylori* 26695 and 128 μg/mL for *H. pylori* J99)^14^ were used as controls. To evaluate the effect of UV exposure on MSI-78A bactericidal performance, growth kinetic assays were also performed using AMP in a solution that was previously exposed to UV LEDs (λ = 365 nm) for 1 min (4 times longer than the exposure time in the microfluidic system).

The CFUs/mL was determined by manually counting the CFUs after 5 days of incubation at 37 °C under microaerophilic conditions.

NP contact with *H. pylori* was studied by TEM. For that, *H. pylori* J99 and *H. pylori* 26695 strains were incubated with either AMP-NP or NorChit-NP for the time required to achieve eradication. To ensure enough bacterial content for posterior TEM analysis, 10 replicates of each condition were combined and centrifuged (3000*g*, RT, 10 min), and the bacterial pellet was fixed with a solution of 2% (v/v) glutaraldehyde, 2.5% (v/v) formaldehyde (both from electron microscopy sciences), and 0.1 M sodium cacodylate buffer (pH 7.4; Merck) for 1 h at RT. The samples were processed as described.^33^

### 2.3 Nanoparticle Cytotoxicity

NP cytotoxicity was evaluated by a direct contact assay in accordance with the international standard ISO 10993-5; 12^34,35^ against two gastric cell lines: the human gastric adenocarcinoma AGS cell line (ATCC CRL-1739), derived from a human stomach adenocarcinoma and well-known for their strong acid secretory function, and the MKN74 (ATCC CRL-2947) cell line, derived from a human gastric carcinoma and widely used for *in vitro* infection models.^13,36^

Cells were grown in the following complete Roswell Park Memorial Institute medium: RPMI 1640 medium with glutamax (Gibco, Invitrogen), supplemented with 10% inactivated FBS (Gibco), 10 U/mL penicillin (Biowest), and 10 μg/mL streptomycin (Biowest), at 37 °C in a humidified atmosphere of 5% CO_2_. For the direct contact assay, cells were seeded for 24 h in 96-well tissue culture polystyrene (TCPS) plates (1 × 10^4^ cells per well). After 24 h, the culture medium was replaced by NP. For that, AMP-NP and NorChit-NP (10^11^ NP/mL) were centrifuged (816 *g*, 10 min, Amicon 50 kDa), suspended in complete RPMI medium, and incubated with cells for another 24 h. AGS and MKN74 cells in fresh media were used as a negative control, whereas cells with 1 mM H_2_O_2_ (Merck) were used as a positive control for the determination of the cytotoxicity profile. Cell metabolic activity was evaluated by a resazurin assay, as described elsewhere,^13^ and general cell morphology after contact with NP was evaluated by cytochemistry.^13^

### 2.4 Statistical Analysis

Statistical analysis was performed using GraphPad Prism Software (GraphPad Software Inc., version 8.0), using a one-way ANOVA or two-way ANOVA, followed by Tukey’s multiple comparisons test. Data were expressed as the mean ± standard deviation (mean ± SD). The mean differences between all analyzed groups were compared, and a *p*-value < 0.05 was considered statistically significant.

## 3 Results and Discussion

### 3.1 Development of Antimicrobial Peptide-Grafted Nanoparticles

#### 3.1.1 AMP and Norbornene Chitosan Synthesis

A modified MSI-78A sequence was synthesized having a flexible 6-aminohexanoic acid (Ahx) spacer and an additional cysteine at the C-terminus (MSI-78A-SH; MW: 2706.6 Da) with ∼90% of purity (determined by HPLC; data not shown), as in previous works.^14,19^

Norbornene functional groups were successfully grafted onto chitosan with a degree of substitution (DS) of 25% (0.25 norbornene groups per chitosan repeating unit). Details regarding DS calculation, using proton nuclear magnetic resonance (^1^H NMR) analysis, are described in the Supporting Information (Figure S1). A DS of 25% is in accordance with what was previously described for chitosan,^37^ using a short reaction time (3 h), with a 1-fold molar excess of carbic anhydride at RT. The higher DS achieved with chitosan in comparison to other norbornene-functionalized polymers (pectin (20%),^28^ and alginate (4–12%)^38^) may be related with the chitosan ability to be functionalized through either its amine or its hydroxyl groups, enhancing the number of available sites for reaction.^21,37,39^

#### 3.1.2 Optimization of AMP-NP Production Settings

##### 3.1.2.1 Microfluidic Chip

The optimized microfluidic chip is represented in Figure 1B. The T-junction microchannel was selected for NorChit-NP production due to its capacity to produce NP with precise size control, narrow size distribution, and reproducibility, in opposition to other geometries such as capillary or coaxial flow reactors.^40^ Its geometry and dimensions were inspired in previous devices designed to produce chitosan NP with sizes ranging from 100 to 200 nm, where chips are 10–60 mm long, with channels 150–200 μm wide and 40–80 μm deep.^22,24−27,41^ Previously described chips were designed for the drug encapsulation in chitosan NP and so, consisting of two inlets for disperse phases (chitosan with drug and usually sodium tripolyphosphate (TPP) solutions) and one outlet to collect the NP.^22,24−27,41^ In this developed microfluidic system, the AMP was grafted onto the surface of previously formed chitosan NP (NorChit-NP). For that, a third inlet was included for AMP injection after NP production in the T-junction (intersection of NorChit with water). Two serpentines were also included to increase the residence time of the NP suspension, leading to improved stability and uniformity.^27^ The second serpentine was designed to increase NP exposure time to the UV light, improving AMP conjugation onto NorChit-NP by the TNPC. In TNPC, thiol groups (present in the AMP) react quickly and efficiently with norbornene units (present in chitosan), triggered by UV light in the presence of an appropriate photoinitiator (VA-086). This click chemistry has been extensively studied for the functionalization of biomaterials (Table 1) and, more recently, for the grafting of an AMP (Dhvar5) onto chitosan hydrogels to prepare thin coatings against Staphylococcus epidermidis (ATCC 35984) and *Pseudomonas aeruginosa* (ATCC 27853).^21,28,37,39^

**Table 1:** Bioconjugation of Antimicrobial Peptides and Proteins onto Biomaterials Using Thiol-Norbornene “Photoclick” Chemistry (TNPC)

biomaterial	conjugated	conjugation yield (%)	results	ref
chitosan	Dhvar5 (AMP)LLLFLLKKRKKRKY	43% for *Nt*-Dhvar5, 30% for *Ct*-Dhvar5	antiadhesive effect: *Staphylococcus epidermidis* (ATCC 35984)	(21)
poly(globalide-*co*-ε-caprolactone)	bovine serum albumin (BSA)	36%	reduction in cellular uptake	(42)
poly(ethylene terephthalate)	HHC10 (AMP) H-KRWWKWIRWNH_2_	0.25%	bactericidal effect: *S. epidermidis* ATCC 32940 & *S. aureus* ATCC 49230	(43)
polyurethane	CGGGREDV (AMP)	32%	promotion of cellular adhesion	(44)

The optimal pH for TNPC is between 4 and 7.^45^ At pH > 9, the reaction yield decreases drastically due to the predominance of thiolate forms, which do not react with norbornene groups.^45^ Although the pH of the injected NorChit solution was ∼3, the mixture with water (pH ∼ 7) and AMP solution (pH ∼ 6.6) in the microfluidic chip rapidly increased the pH values adequate for AMP grafting onto NorChit-NP (pH ∼ 7).

Overall, this is an ecofriendly and sustainable system, as the device uses minimal consumption of reagents and can be reused multiple times; moreover, by allowing the integration of multiple steps into a single device, there is no need for multiple equipment, which reduces time and energy consumption. Nevertheless, in future studies, PDMS could be replaced by a more environmentally friendly polymer and there are some promising alternatives already reported.^46^

##### 3.1.2.2 NorChit-NP

**NP size and concentration: Effect of NorChit concentration:** The effect of NorChit concentration (1.5, 2, and 2.5 mg/mL) in NP size and concentration was determined by NTA. Results in Table 2 showed that an increase in NorChit concentration led to a small decrease in NP size (no significant differences) without affecting their concentration (∼2 × 10^11^ NP/mL). Therefore, NorChit-NP was further produced using the less concentrated NorChit solution (1.5 mg/mL) in line with more sustainable research and envisioning a possible tech transfer scenario.

**Table 2:** Size (nm), D90 (nm), and Concentration of NorChit-NP Produced with Different NorChit Concentrations (1.5, 2, and 2.5 mg/mL) Determined by NTA^a^

NorChit solution	NorChit-NP		
concentration (mg/mL)	size (nm)	D90 (nm)	concentration (10^11^ NP/mL)
1.5	107 ± 55	164	2.2 ± 0.2
2.0	83 ± 54	119	2.8 ± 0.4
2.5	81 ± 51	128	2.7 ± 0.3

a D90: the portion of the NP with diameters below this value is 90%.

**NP Stability in SGF: Effect of cross-Linker:** To evaluate the need of a cross-linker, NP produced (i) with a cross-linker (dithiothreitol; DTT) and exposure to UV light (λ = 365 nm; 5 min) and (ii) without a cross-linker were incubated in SGF (pH 1.2). NP stability was assessed by alterations in their concentration (associated with NP dissolution), size (swelling and/or aggregation), and zeta potential (stability over time).

Results (Table 3) showed no differences between the concentration and size of NP produced with and without the cross-linking step after immersion in SGF. The zeta potential was also maintained over time in the NorChit-NP, without DTT (no significant differences). These results demonstrated that the use of a cross-linker was not required during NorChit-NP production. This is a huge advantage since, in addition to being easier to produce, these NP allow them to thwart toxicity issues usually associated with cross-linkers.^47^ The NP stability in SGF without cross-linking may be related with the interaction between the hydrophobic norbornene groups. During NorChit-NP formation, the hydrophobic norbornene groups are repelled from the surrounding water molecules and tend to form internal hydrophobic clusters.^48^ This leads to a decrease in the overall interfacial free energy of the system (by reducing the overall surface area of the hydrophobic groups) and, consequently, to higher NP stability.^49^

**Table 3:** Stability of NorChit-NP with and without Cross-Linking in Simulated Gastric Fluid (SGF, pH 1.2)^a^

	without DTT			with DTT	
time (h)	[NorChit-NP] (*x* 10^11^ NP/mL)	size (nm)	zeta potential (mV)	[NorChit-NP] (*x*10^11^ NP/mL)	size (nm)
0	7.4 ± 0.3	107 ± 55	6 ± 3	7.4 ± 0.1	122 ± 43
3	4.1 ± 0.6	103 ± 35	–2 ± 3	3.9 ± 0.2	102 ± 39
120	2.8 ± 0.4	123 ± 72	–2 ± 9	2.7 ± 0.5	120 ± 47

a The cross-linking occurred in the presence of a dithiolated cross-linker (dithiothreitol, DTT) through TNPC reaction (5 min, UV light λ = 365 nm).

##### 3.1.2.3 AMP Bioconjugation onto NorChit-NP: Effect of UV Light Intensity

Since UV light intensity can influence TNPC efficiency,^21,28^ the effect of UV light intensity on AMP bioconjugation onto NorChit-NP was optimized using different LED combinations (Figure S3) and a FITC tag model peptide mixed with the photoinitiator VA-086. Results are presented in Table 4.

**Table 4:** Effect of UV Light (LED; λ = 365 nm) Intensity and Peptide Concentration on Peptide Grafting Yields onto NorChit-NP via TNPC^a^

grafting yield (%)			
[model peptide] (mg/mL)	**1 LED** (35 mW/cm^2^)	2 LEDs in parallel (110 mW/cm^2^) superior: 35 mW/cm^2^ inferior: 75 mW/cm^2^	3 LEDs in series (105 mW/cm^2^) 35 mW/cm^2^ thrice
0.25	43	64	61
0.50	50	74	64

a Quantification was performed using a fluorescent model peptide (CGGGGRGDSP; with a fluorescein isothiocyanate (FITC) tag). The reaction yield was measured indirectly by measuring the peptide-FITC levels (excitation wavelength of 485 nm and emission wavelength of 528 nm, Synergy Mx) in the peptide solution before and after reaction with NorChit-NP.

Table 4 shows that the yield of peptide grafting by TNPC increases with an increase in light intensity. Indeed, a yield of 74% was obtained using two parallel LEDs (one on each side of the microfluidic system; 110 mW/cm^2^) and 0.5 mg/mL of peptide. The grafting yield was higher for 0.5 mg/mL of AMP.

For the TNPC grafting reaction, the presence of a photoinitiator (VA-086) was previously studied.^21^ UV light with a higher intensity (110 mW/cm^2^) can favor the grafting reaction by providing more energy to the photoinitiator and increasing the concentration of reactive species (free radicals, cations, and anions). The TNPC reaction is also dependent on the norbornene groups available and the concentration of the thiolated compound to be conjugated. Although the amount of norbornene groups on chitosan was calculated by ^1^H NMR, the percentage of norbornene groups that are exposed to the NP is unknown. Nevertheless, the AMP grafting yield (Table 4) demonstrated that several groups were available for bioconjugation.

To evaluate the effect of AMP concentration, AMP-NP was produced using different MSI-78A-SH concentrations (0.25, 0.5, and 1 mg/mL). The efficacy of the produced AMP-NP was indirectly evaluated by its bactericidal activity against *H. pylori* J99. Results demonstrated that 0.5 mg/mL MSI-78A-SH was enough to obtain bactericidal AMP-NP (details in Supporting Information, Figure S4).

#### 3.1.3 AMP-NP Characterization

**Figure 2:**
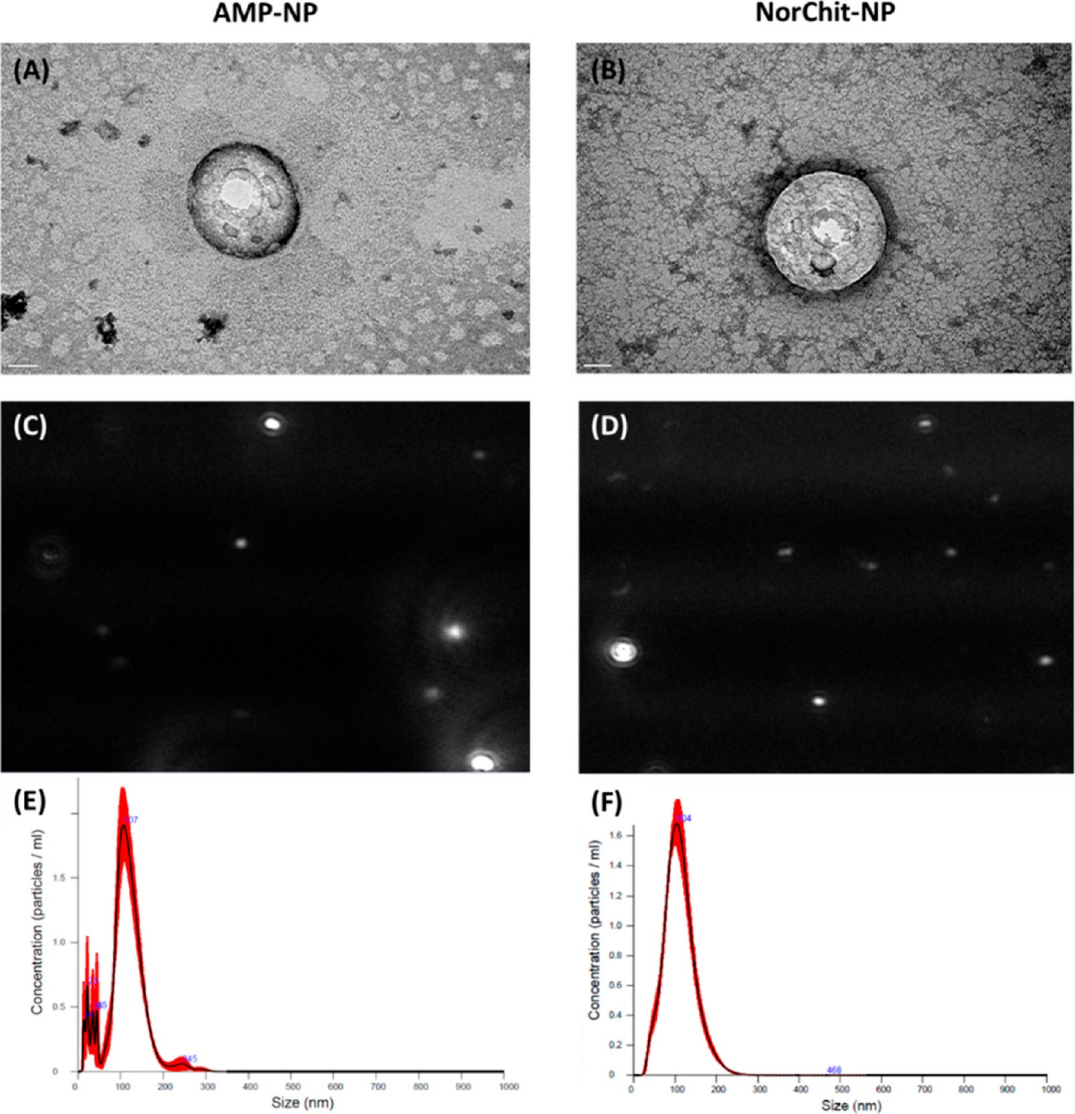
Negative staining TEM micrographs of (A) AMP-NP and (B) NorChit-NP (scale bars: 50 nm). Representative NTA video frame of (C) AMP-NP and (D) NorChit-NP and respective NP distribution among concentration and size (E) AMP-NP and (F) NorChit-NP.

Optimized NP (NorChit-NP and AMP-NP), prepared as described in Figure 1 and Section 2.1.2, was characterized regarding size and concentration using TEM, NTA, and DLS. Figure 2 shows that all the NP have a spherical shape and did not aggregate after production. The absence of NP precipitation can be explained by (i) the use of water to interrupt the flow of NorChit solution and produce single NP, (ii) the protonation of chitosan and AMP primary amine groups in acidic pH, or (iii) the absence of a cross-linker. Moreover, AMP grafting onto NorChit-NP did not affect the NP morphology (Figure 2A,B). NorChit-NP and AMP-NP had an average size of 107 ± 55 and 113 ± 43 nm, respectively (Table 5). Concerning the concentration, no significant differences were observed between NP type (Table 5) and among batches (*n* = 3), confirming the reproducibility of the designed microfluidic device. NP distribution (size and concentration) is shown in Figure 2E,F. Regarding surface charge, the AMP-NP presented a higher positive zeta potential value than NorChit-NP. This was expected due to the cationic nature of the AMP.

**Table 5:** Size, Mode, and D90 (nm); Concentration of NP (*x* 10^11^ NP/mL) and Zeta Potential (mV). The Final Volume of Each NP Batch Was 1.5 mL, Which Corresponds to the NorChit Final Concentration of 4.7% (v/v)

NP	size (nm)	mode (nm)	D90 (nm)	concentration (10^11^ NP/mL)	zeta potential (mV)
AMP-NP	113 ± 43	107	157	2.1 ± 0.1	14.3 ± 7
NorChit-NP	107 ± 55	93	164	2.2 ± 0.2	5.9 ± 3

**Figure 3:**
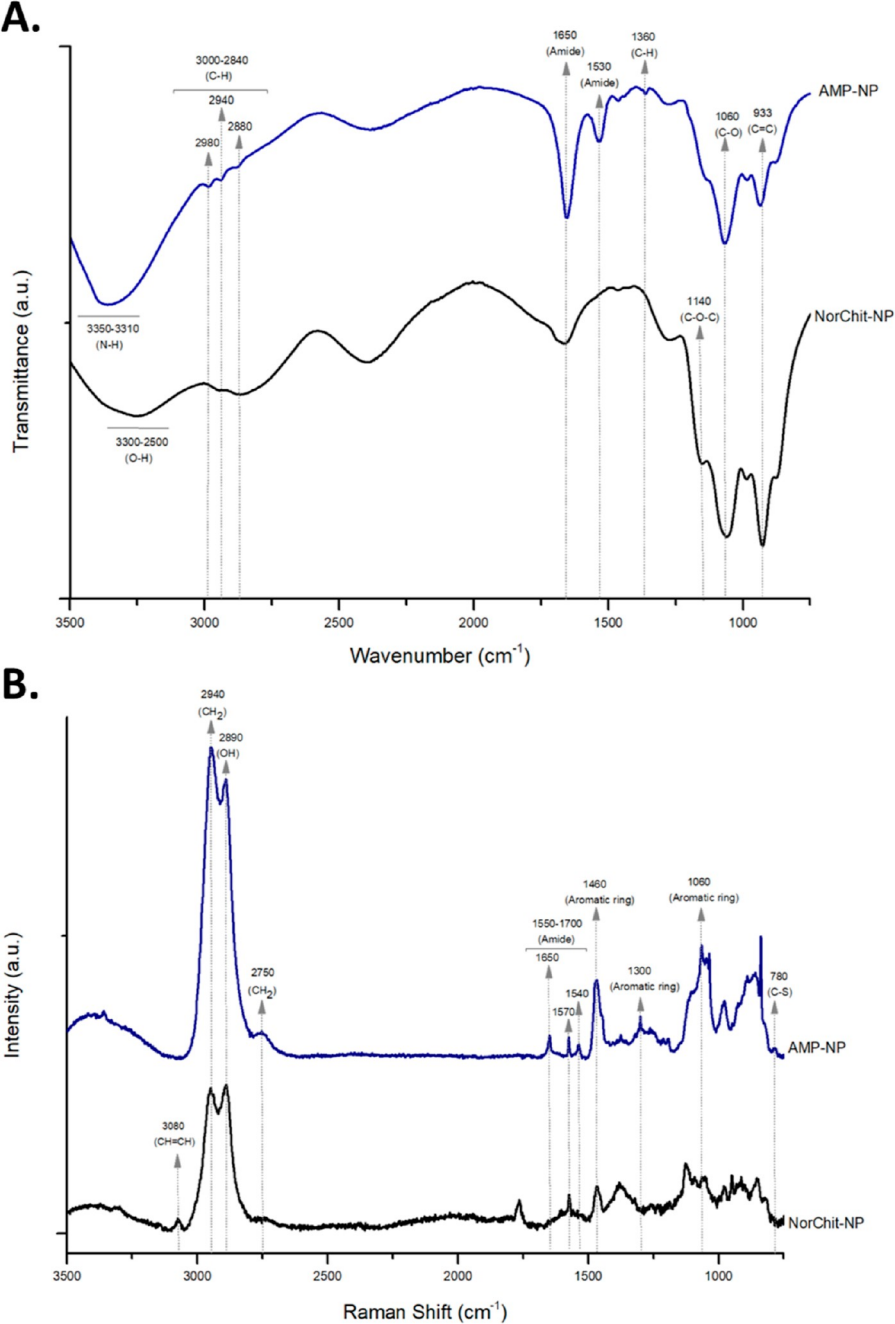
(A) Fourier-transform infrared spectroscopy (FTIR) and (B) Raman spectroscopy spectra of NorChit-NP and AMP-NP, in the 3500–750 cm^–1^ region.

FTIR and Raman spectra of AMP-NP and NorChit-NP are shown in Figure 3A,B, respectively.

The FTIR spectrum of NorChit-NP indicates a broad peak in the 1760–1650 cm^–1^ region, which can be assigned to the carbonyl (C=O) and amide (N–C==O) groups from the norbornene moiety grafted to chitosan.^50^ The FTIR spectrum obtained for AMP-NP shows an increase of the intensity of the peak at 1660 cm^–1^ (amide I from the peptide) as compared to the intensity of the peak at 1060 cm^–1^ (characteristic of the chitosan ether groups, C–O–C). The ratio between the height of amide I (1660 cm^–1^) and ether (1060 cm^–1^) peak was quantified as described elsewhere.^19,51^ The higher peak ratio was obtained for AMP-NP (1.2) compared with NorChit-NP (0.4). These results, combined with the reduction of the 933 cm^–1^ peak (C=C) and the appearance of new peaks at (i) 1530 cm^–1^ (amide II band in peptides), (ii) 2840–3000 cm^–1^ and 1360 (C–H), (iii) 3355 (N–H) indicated the successful grafting of AMP onto the NorChit-NP. Raman spectrum (Figure 3B) reinforced the effective graft of AMP by the disappearance of the 3080 cm^–1^ peak (CH=CH) combined with the appearance of new AMP bound characteristics peaks: (i) 1540 cm^–1^ (amide II), (ii) 1650 cm^–1^ (amide I), (iii) 2750 and 2940 cm^–1^ (CH_2_), (iv) 1060, 1300, and 1460 cm^–1^ (aromatic rings of phenylalanine), and (v) 780 cm^–1^ (C–S).^52^

AMP grafted onto the NorChit-NP was quantified directly by AAA. Results demonstrated that in the tested conditions, the AMP grafting yield was 40%, corresponding to 4.6 × 10^–10^ μg of AMP grafted per each NP and to 96 μg of AMP per batch of AMP-NP used on the antibacterial performance assays (10^11^ NP) (Table 6). The obtained yield was in accordance with the literature for chitosan functionalization using TNPC.^21,44^ Concerning AMP adsorption onto NorChit-NP, the maximum amount of AMP adsorbed was residual (∼4%), highlighting that most of the AMP present in the sample was effectively immobilized. Table 6 also shows the theoretical and experimental ratios of the amino acids presented in the peptide per se and in AMP-NP. According to the results obtained, the peptide amino acid sequence remained stable during the TNPC. Overall, these results confirm TNPC as a high yield (high amount of immobilized AMP) and fast (seconds or a few minutes) peptide grafting method.

**Table 6:** AAA of AMP (Sequence: GIGKFLKKAKKFAKAFVKILKK-Ahx-C; MW: 2706.6 Da) Grafted or Adsorbed onto NP^a^

nmol (hydrolyzed conjugated)					experimental ratio	
amino acid		AMP-NP immobilized	AMP-NP adsorbed	theoretical ratio	AMP-NP immobilized	AMP-NP adsorbed
glycine^c^	G	75.7	12.3	2	2	3
alanine	A	86.5	15.2	3	2	4
valine^c^^d^	V	31.2	3.7	1	1	1
isoleucine^c^	I	68.4	10.7	2	2	3
leucine^c^	L	64.9	8.6	2	2	2
phenylalanine^c^^d^	F	115.5	10.9	3	3	3
lysine^d^	K	237.1	33.2	9	7	9
AMP content^b^		35.6	3.7			
AMP mass (μg AMP/10^11^ NP)		96	9.9			

a The AMP content was calculated by the average through the ratios of each hydrolyzed AA (nmol) considered for the calculation and the respective experimental ratio. The AMP mass corresponds to the final AMP-content (μmol) *x* MW_AMP_ (Da).

b nmols/residue, estimated.

c Amino acids are considered for the calculation of immobilized.

d Amino acids are considered for the calculation of the adsorbed peptide quantity.

#### 3.1.4 Antibacterial Properties and Cytotoxicity

##### 3.1.4.1 Bactericidal Effect

*H. pylori* infection is usually multistrain, which strongly contributes to the failure of eradication therapy.^53^*H. pylori* 26695 and *H. pylori* J99 are highly pathogenic clinical isolates.^54,55^ AMP-NP bactericidal performance was evaluated using these two *H. pylori* strains by performing growth kinetics assays in the presence of AMP-NP (∼10^11^ NP/mL, 96 μg/mL). AMP (MSI-78A), AMP exposed to UV LEDs (λ = 365 nm) during 1 min, and NorChit-NP and pure *H. pylori* culture (with no contact with NP) were used as controls.

**Figure 4:**
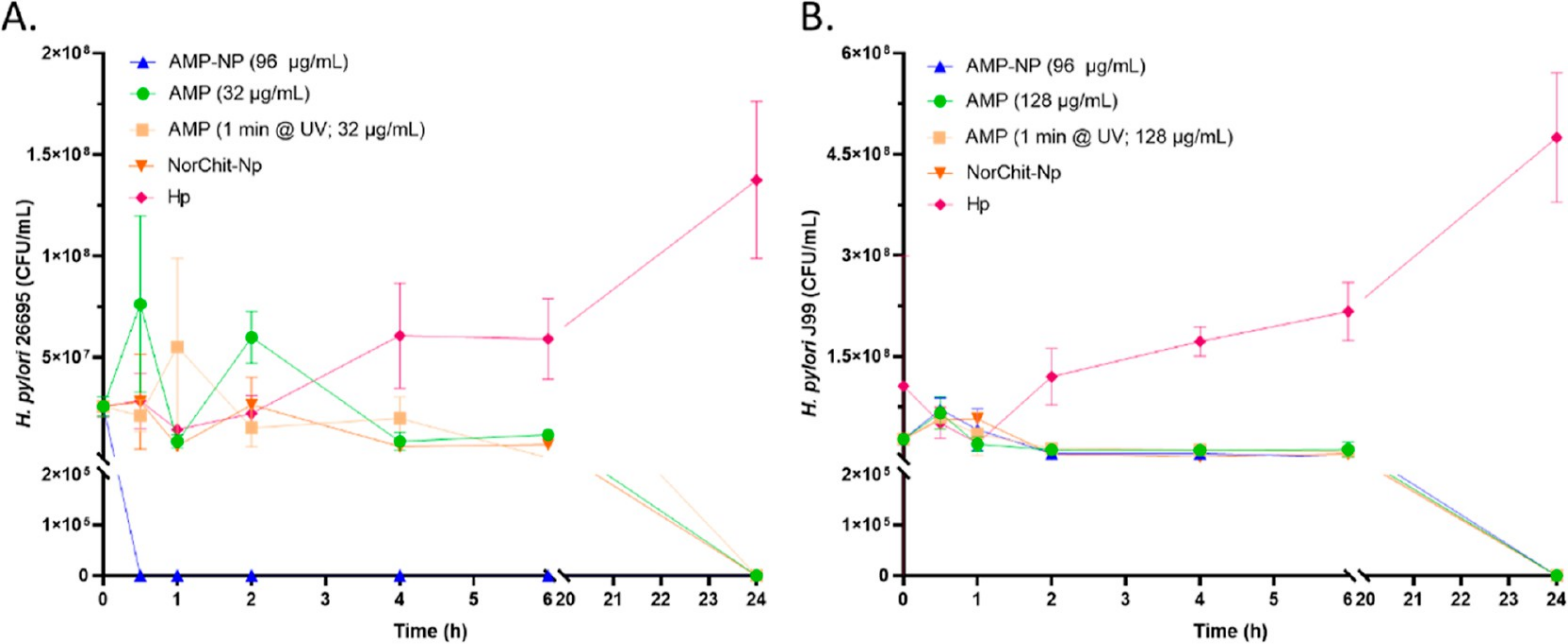
Time-kill assay of AMP-NP against *H. pylori* (A) 26695 (MBC = 32 μg/mL) and (B) J99 strains (MBC = 128 μg/mL). AMP in solution, AMP in solution exposed to UV light for 1 min, and NorChit-NP and pure bacterial culture were used as controls. Assays were performed in Brucella Broth supplemented with 10% FBS (three independent experiments with duplicates).

**Figure 5:**
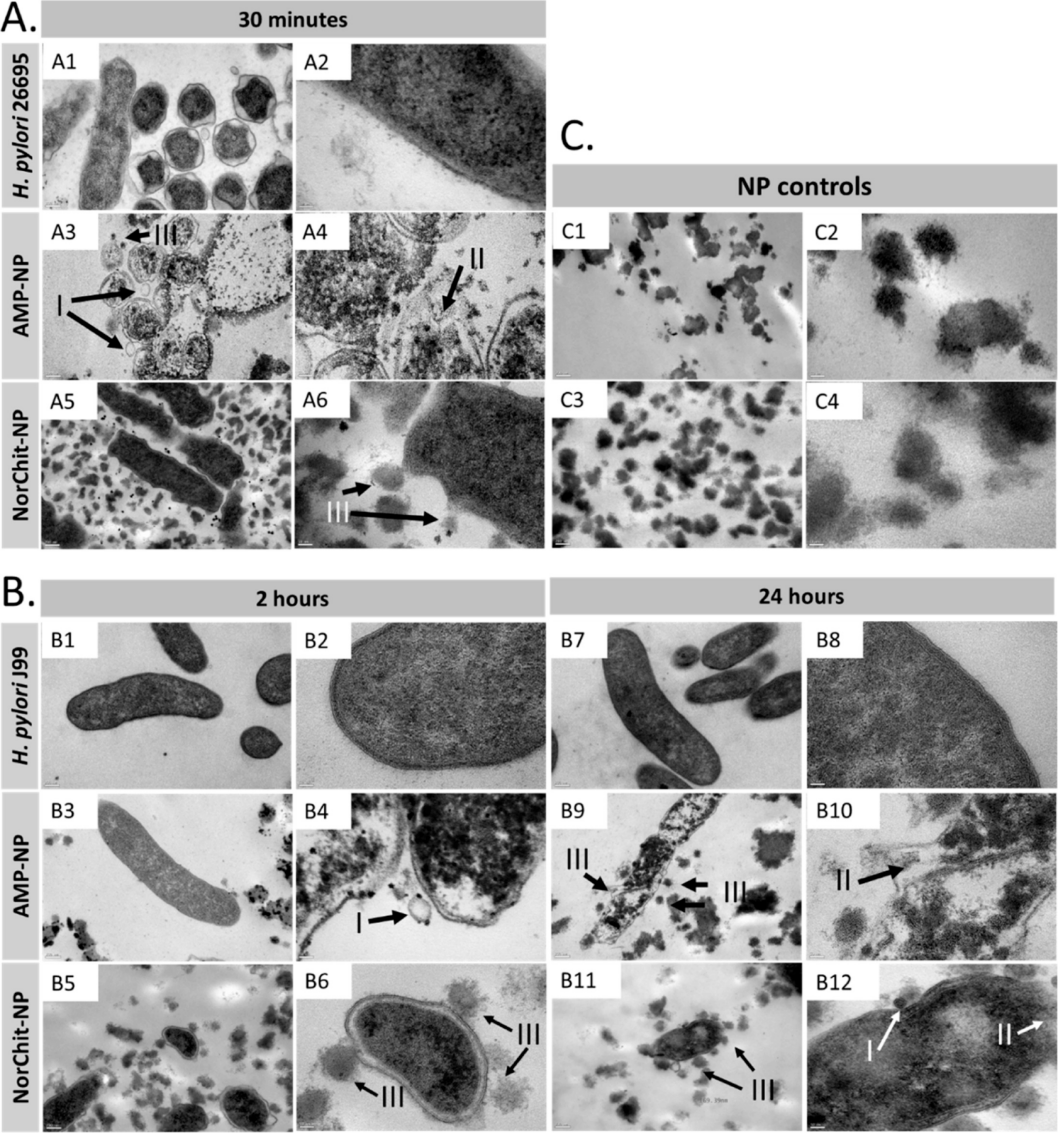
TEM micrographs of the NP. (A) Incubated with *H. pylori* 26695 strain for 30 min and (B) *H. pylori* J99 strain for 2 and 24 h. (C) NP controls (without bacteria). Scale bars: 200 nm (A1,A3,A5,B1,B3,B5,B7,B9,B11,C1,C3) and 50 nm (A2,A4,A6,B2,B4,B6,B8,B10,B12,C2,C4). Arrows indicate (I) vesicle formation; (II) release of cytoplasm; and (III) NP contact with bacteria.

AMP-NP (10^11^ NP/mL with 96 μg/mL AMP) showed a bactericidal effect against both *H. pylori* strains. However, the bactericidal effect was faster for the *H. pylori* 26695 (30 min) than for the *H. pylori* J99 strain (24 h), as demonstrated in Figure 4A,B, respectively. Lower AMP-NP concentrations (10^9^ and 10^10^ NP/mL) did not have a bactericidal effect against this gastric pathogen (data not shown). The faster AMP-NP effect against *H. pylori* 26695 can be explained by the lower MBC of MSI-78A in solution (32 μg/mL) for this strain, as compared to *H. pylori* J99 (128 μg/mL).^14^ The grafted peptide concentration in AMP-NP (∼10^11^ NP/mL) was 96 μg/mL, which is 3 times higher than the MBC for *H. pylori* 26695, but lower than the MBC established for *H. pylori* J99, suggesting an enhanced AMP bactericidal activity after grafting onto NorChit-NP. The rapid bacterial eradication process anticipates an irreversible effect with a low propensity for the development of a resistance mechanism, especially for the *H. pylori* 26695 strain that was eradicated after only 30 min of exposure to AMP-NP. Conversely to the previous work, where AMP was encapsulated in chitosan/alginate NP,^11^ here the AMP was grafted onto the NP surface. Therefore, it is expected to achieve faster eradication since there is no need for NP degradation to release the AMP. Results in Figure 5 suggest a contact-killing mechanism as previously described for surface immobilized AMP.^19^ Moreover, a significant improvement on AMP activity after grafting onto NorChit-NP was demonstrated when compared to chitosan microspheres (AMP-ChMic).^19^ AMP-ChMic was bactericidal against *H. pylori* J99 after 6 h but required 3 times more AMP (277 μg per batch) than the concentration used in this study (96 μg per batch). Furthermore, to obtain this bactericidal effect with the microspheres, a long heterobifunctional spacer maleimide polyethylene glycol succinimidyl carboxymethyl ester (NHS-PEG_113_-MAL) was needed to better expose the AMP to the bacteria,^19^ whereas in AMP-NP no spacers were used.

In solution, the AMP at their minimum bactericidal concentration (MBC: 32 μg/mL for *H. pylori* 26695 and 128 μg/mL for *H. pylori* J99^14^) led to both *H. pylori* strains eradication after 24 h (Figure 4A,B). Moreover, the exposure to UV light did not affect the MSI-78A bactericidal activity against both *H. pylori* strains (Figure 4A,B), suggesting that the remaining unreacted AMP (Section 2.1.2) can be recovered, freeze-dried, and repurified for further use.

Results also demonstrated that NorChit-NP (∼10^11^ NP/mL), used as a control, was bactericidal against both *H. pylori* strains after 24 h of incubation (Figure 4A,B). This fact could be related to the low DA of the chitosan used in this study (2–3%). It was previously described that chitosan with lower DA (higher levels of free amines) exhibits stronger antibacterial activity, which was explained by electrostatic interactions between the protonated amine groups of chitosan and the negatively charged bacteria membrane.^56^ Chitosan NP (70–120 nm), produced using chitosan with 5% DA, were able to eradicate *H. pylori* infection in 75% of infected mice.^12^ Besides their killing effect, which was linked to cytoplasmatic content release due to alterations in *H. pylori* membranes, these NP were also able to permeate the bacterium membranes due to their nanometric size, interfering with bacteria metabolism. In another *in vivo* study, chitosan microspheres (40 or 150 μm) developed for binding and removing *H. pylori* from infected stomach were more efficient in reducing *H. pylori* load when prepared with chitosan with lower DA (6% *versus* 16%).^13^ Besides DA, size and Ch functionalization may also play a role in the final antibacterial performance. Importantly, in our previous work, chitosan microspheres (4 μm) prepared with the same chitosan described here were able to bind the bacteria but did not kill *H. pyloriin vitro*,^19^ suggesting that the bactericidal effect of chitosan particles is also related to their size. In addition, the chitosan used was functionalized with norbornene groups, which may lead to the replacement of electrostatic interactions by hydrophobic interactions. Indeed, the bactericidal activity of norbornene groups (in solution) was previously reported against *Escherichia coli* D31 and *Bacillus subtilis* ATCC 8037, due to the high hydrophobicity of norbornene groups and their stronger capacity to interact with the inner core of bacterial membranes, causing their destabilization.^57^ Hence, our results suggest that the bactericidal effect of NorChit-NP was related with a possible synergistic effect of exposed norbornene groups, low chitosan DA, and particle nanometric size.

The contact of NP with *H. pylori* 26695 over 30 min and with *H. pylori* J99 over 24 h (the time required to achieve bacterial eradication) was observed by using TEM. An intermediate time (2 h) was also used for *H. pylori* J99 to analyze the effect of NP onto the bacterial cells prior to death.

TEM images show that pure bacterial culture (Figure 5A1,A2,B1,B2,B7,B8) had a normal morphology, presenting intact cell membranes. Nevertheless, *H. pylori* 26695 (Figure 5A1,A2) had a more fragile appearance than *H. pylori* J99 (Figure 5B1,B2,B7,B8), with some spaces in the intracellular content observed. This typical aspect of *H. pylori* 26695 was previously observed by Correia *et al*.^58^ As a comparative control, Figure 5C shows NorChit-NP and AMP-NP alone (i.e., without bacteria) after incubation with supplemented media for 24 h and subjected to histological sectioning (as described in Section 2.2.2).

After incubation, contact of the NP with bacteria was easily observed in all images (arrow III, Figure 5). This effect was probably due to electrostatic attraction between the anionic membrane of *H. pylori* and the cationic nature of both chitosans. Figure 5 (arrow III) also shows that each *H. pylori* was exposed to a multitude of NP, since a high ratio of NP/bacteria was used (10^11^ NP for ∼10^7^ bacteria).

AMP-NP led to bacterial membrane destabilization, the formation of extracellular vesicles, and the release of the cytoplasmatic content. This behavior was expected since peptides of the MSI-78 family, in solution, interact with bacterial membranes (*E. coli*), either by promoting their disruption or formation of toroidal pores.^59,60^ This AMP-NP effect was observed at the killing time (Figure 5 A3,A4,B9,B10) and after 2 h incubation (*H. pylori* J99) with AMP-NP (Figure 5B3,B4).

NorChit-NP contact with bacteria was also observed (Figure 5A5,A6,B5,B6,B11,B12). However, although some vesicle formation and possible instability in the membrane of the bacteria were observed (Figure 5A6,B12), bacteria incubated in the presence of NorChit-NP had a denser and more uniform cytoplasm than bacteria exposed to AMP-NP. A similar effect was observed in *P. aeruginosa* treated with chitosan-polyethylene glycol-peptide conjugate, in which the cytoplasmic material was agglomerated by the flocculation of chitosan after the conjugate entered the bacteria.^61^ Besides, it was previously described that the bactericidal capacity of norbornene groups was related with the destabilization of bacterial membranes.^57^

#### 3.1.5 Cytotoxicity

**Figure 6:**
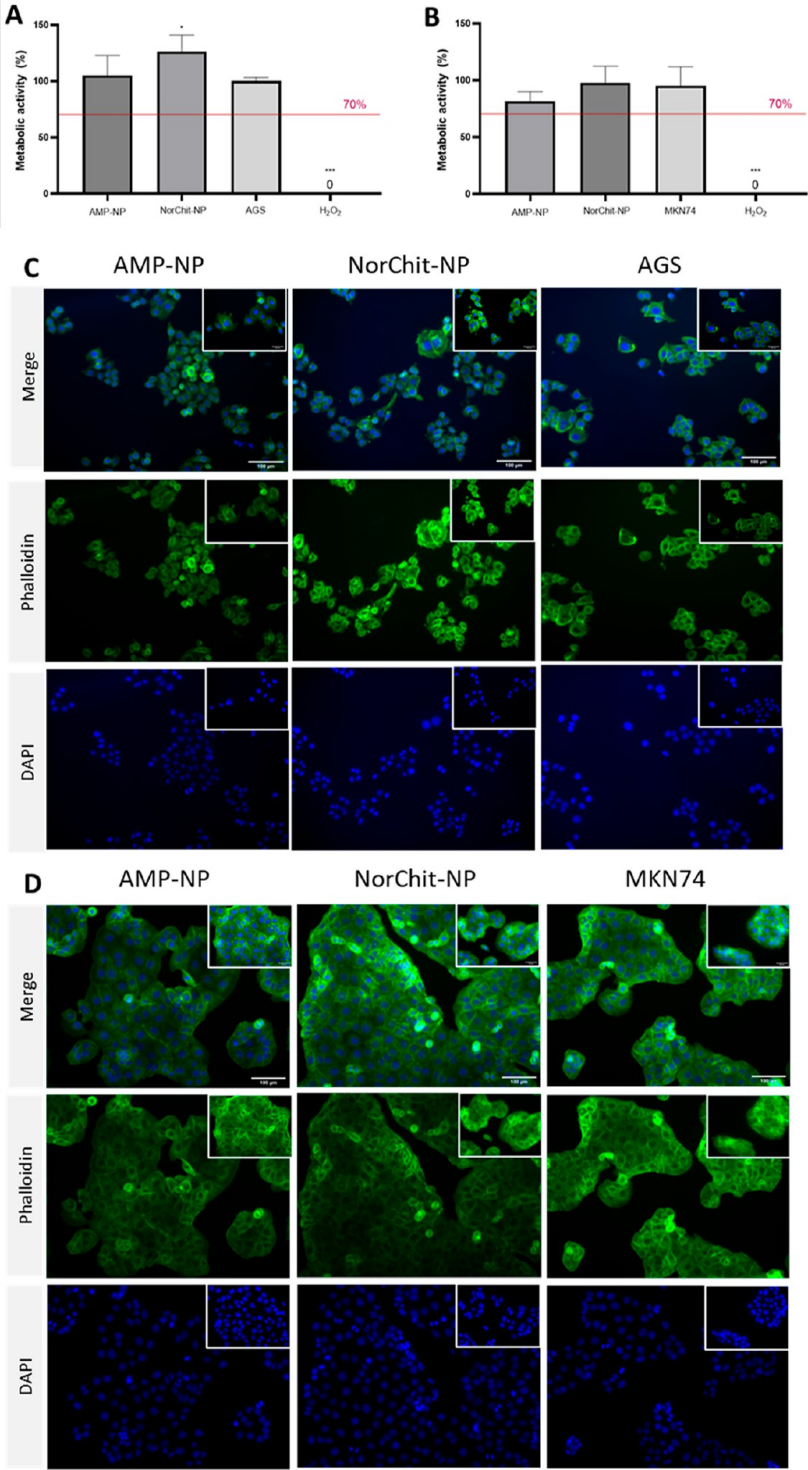
Metabolic activity of (A) AGS and (B) MKN74 cell lines after direct contact with NP (direct contact, ISO 10993-5; 12). Cell metabolic activity was assessed by the resazurin assay after 24 h incubation with NP (NorChit-NP and AMP-NP), no treated cells (TCPE; negative control), or 1 mM 30% V H_2_O_2_ (positive control). Metabolic activity is expressed as the percentage of the cell metabolic activity of treated cells in relation to cells in culture medium only. *NorChit-NP is significantly different from cells in culture medium only; *** positive control is significantly different from all other samples (one-way ANOVA, followed by Tukey’s multiple comparison test, *p* < 0.05). (C) AGS and (D) MKN74 morphology were analyzed by cytochemistry: cells were stained with DAPI (nucleus)—blue and phalloidin (F-actin in cytoskeleton)—green. Fluorescence images were taken by IFM after contact with NP. Cells cultured in the presence of H_2_O_2_ died and therefore detached from the bottom of the plate, the reason why no cells were seen after staining. Magnifications = 200× and 400×; scale bar = 100 μm for background image and 50 μm for inserts.

The effect of AMP-NP and NorChit-NP on cell metabolic activity is described in Figure 6A for AGS cells and in Figure 6B for MKN74 cells. AMP-NP and NorChit-NP, at the highest concentration tested (10^11^ NP/mL, 96 μg/mL AMP), were cytocompatible to both cell lines according to the ISO standard 10993-5; 12,^34,35^ since the metabolic activity values were above the 70% threshold (Figure 6A,B). As expected, cells exposed to H_2_O_2_ showed extensive cell lysis and vacuolization, with less than 1% of the cells metabolically active.

Moreover, gastric cells retained regular morphology (epithelial shape) across all of the conditions tested (NorChit-NP and AMP-NP, 10^11^ NP/mL), forming a monolayer (Figure 6C,D). AGS (Figure 6C) is a cell line exhibiting epithelial morphology with a polygonal shape (mushroom-like) that, in healthy conditions, demonstrated elongation and the formation of a monolayer. MKN74 cells (Figure 6D) also have an epithelial morphology but may appear more flattened and have visible cytoplasmic extensions, represented by a more intense green color. The negative control (only cells) and the conditions in contact with NP reveal similar morphologies and confluency, highlighting that the NP did not negatively affect the cells. These results reinforce the cytocompatible profile of NP at the bactericidal concentration.

#### 3.1.6 Conclusions

A simple, versatile, and time- and cost-effective microfluidic system to produce AMP-grafted NP was designed and optimized. The main advantage of the herein designed system is the possibility to simultaneously produce, cross-link, and conjugate chitosan NP with thiolated peptides using a single device. Importantly, this system can be adapted for the bioconjugation of other thiolated ligands into any norbornene-modified polymeric NP.

The potential of the designed “one pot” microfluidic system was demonstrated using a thiolated AMP (MSI-78A-SH) with antibacterial performance against *H. pylori* grafted onto norbornene-chitosan NP (AMP-NP). AMP-NP, stable in acidic conditions, was bactericidal against two highly pathogenic *H. pylori* strains, without cytotoxic effects to gastric cell lines at the bactericidal concentration. Moreover, due to its quick and effective *H. pylori* eradication process, this AMP-NP could overcome antibiotic overuse and prevent the rise of antimicrobial resistance, a major public health challenge.

## Supporting Information Available

^1^H NMR, FTIR, UV-LED conformations, and bactericidal effect of AMP-NP against *H. pylori* J99 strain as gastric bacteria model (PDF)

## Abbreviations

AAA: amino acid analysis
AMPs: antimicrobial peptides
BA: blood agar
BB: Brucella broth
CagA: cytotoxin-associated gene A
ChMic: chitosan microspheres
CFUs: colony forming units
DMF: *N*,*N*-dimethylformamide
DS: degree of substitution
DTT: dithiothreitol
FTIR: Fourier-transform infrared spectroscopy
^1^H NMR: proton nuclear magnetic resonance
NP: nanoparticles
NTA: nanoparticle tracking analysis
NorChit: norbornene-chitosan
PBS: phosphate-buffered saline
PDMS: poly dimethylsiloxane
rpm: rotations per minute
RT: room temperature
SGF: simulated gastric fluid
TEM: transmission electron microscopy
TNPC: thiol–norbornene “photoclick” chemistry
TSA: tryptic soy agar
TPP: sodium tripolyphosphate
UV: ultraviolet
VA-086: 2,2′-azobis[2-methyl-*N*-(2-hydroxyethyl)propionamide
VacA: vacuolating cytotoxin A
