# Supplementary material for: One-Pot Microfluidics to Engineer Chitosan Nanoparticles Conjugated with Antimicrobial Peptides Using “Photoclick” Chemistry: Validation Using the Gastric Bacterium Helicobacter pylori
Source: ACS Appl Mater Interfaces. 2024 Mar 14;16(12):14533–47. doi: 10.1021/acsami.3c18772 (PMC10982938; doi:10.1021/acsami.3c18772)
Supplement: Supplementary file 1 — am3c18772_si_001.pdf [file am3c18772_si_001.pdf]

Supporting Information

One-pot microfluidics to engineer chitosan nanoparticles conjugated with antimicrobial peptides using "photoclick" chemistry: validation using the gastric bacterium *Helicobacter pylori*

Diana R. Fonseca[1,2,3], Pedro M. Alves[1,2,3,4], Estrela Neto[1,2], Beatriz Custódio[1,2,5], Sofia Guimarães[1,2], Duarte Moura[1,2,3], Francesca Annis[1,2], Marco Martins[6], Ana Gomes[4], Cátia Teixeira[4], Paula Gomes[4], Rúben F. Pereira[1,2,5], Paulo Freitas[6,7], Paula Parreira[1,2], M. Cristina L. Martins[1,2,5]*

[1]i3S – Instituto de Investigação e Inovação em Saúde, Universidade do Porto, Rua Alfredo Allen, 208, 4200-135, Porto, Portugal

[2] Instituto Nacional de Engenharia Biomédica, Universidade do Porto, R. Alfredo Allen 208, 4200-135, Porto, Portugal

[3]Faculdade de Engenharia, Departamento de Engenharia Metalúrgica e de Materiais, Universidade do Porto, R. Dr. Roberto Frias, 4200-465 Porto, Portugal

[4]LAQV-REQUIMTE, Departamento de Química e Bioquímica, Faculdade de Ciências, Universidade do Porto, Rua do Campo Alegre 685, 4169-007, Porto, Portugal

[5]ICBAS—Instituto de Ciências Biomédicas Abel Salazar, Universidade do Porto, Rua de Jorge Viterbo Ferreira, 4050-313 Porto, Portugal

[6]INL, International Iberian Nanotechnology Laboratory, Av. Mte. José Veiga s/n, 4715-330, Braga, Portugal

[7]INESC-MN, INESC Microsystems and Nanotechnologies, Rua Alves Redol 9,1000-029, Lisboa, Portugal

* Corresponding author: cmartins@ineb.up.pt

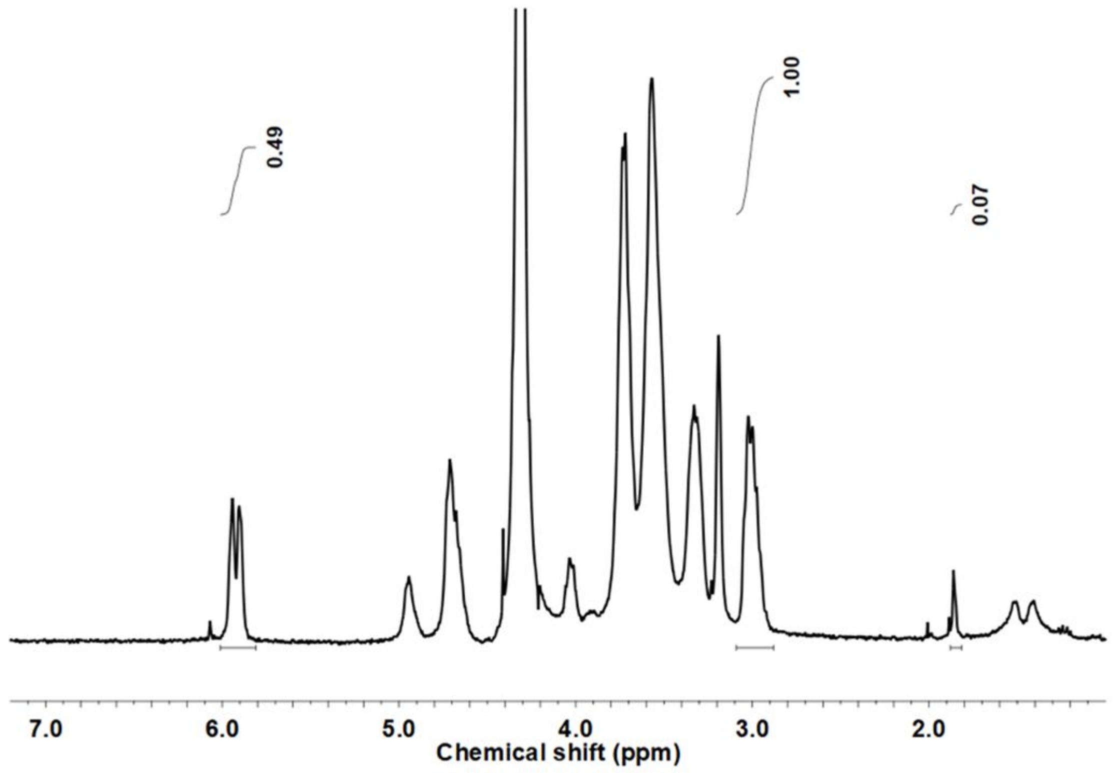

$$(\mathbf{A})\ Degree\ of\ substituion\ (DS) = \frac{\int HC = CH\ from\ CA}{\int H - 1\ from\ Ch} x\ \frac{1}{2}$$

$$(\mathbf{B})\ Degree\ of\ acetylation\ (DA) = \frac{\int 1.81\ ppm}{\int 3.00\ ppm} x\ \frac{1}{3}$$

Figure S1. Determination of (a) the NorChit degree of substitution by ^{1}H NMR (400 MHz, 0.5 M DCl in D_2O, 70 °C), applying Eq. **A**, by comparing the integral of the peaks of norbornene vinyl protons (2-H, to that of the peak from the H-2 proton of chitosan repeating units after proton nuclear magnetic resonance (NMR) analysis [1,2]. For integration, peak from the H-2 proton of chitosan was used as a reference. ∫*HC=CH from CA* is the integral of alkene peaks from norbornene and ∫*H-1 from Ch* is the integral of the H-2 proton of chitosan, i.e., **DS = (0.49)/1.00 x ½ = 0.25.** (b) Determination of Degree of Acetylation (DA) applying the Eq.**B**. The integral of the peak at 1.81 ppm (from the 3 proton of the acetyl group) was compared with the one of the peak at 3.00 ppm (H-2 proton). A factor of 1/3 was applied to the equation for proton correction in the contribution of each peak, i.e., **DA(NMR) = (0.07)/1.00 x 1/3=0.023=2.3%**

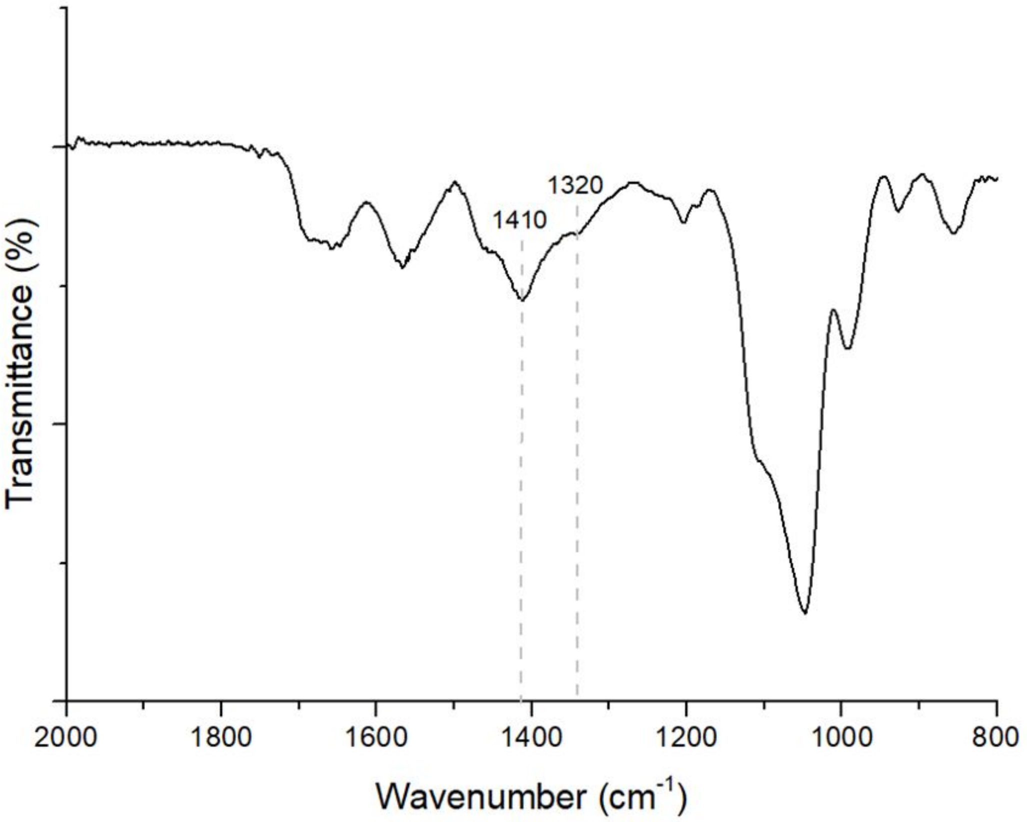

$$\textbf{(C)}\ DA\ (FTIR) = \left(\frac{A\ 1320}{A\ 1420} - 0.3822\right)/0.0311\ = 2.9\%$$

Figure S2. Fourier-transform infrared spectroscopy (FTIR) spectra of NorChit-NP, in the 2000 cm^{-1}- 800 cm^{-1} region. DA was obtained applying the Brugnerotto equation, using the FTIR peaks (Eq. **C**) [3]. A_{1320} is the characteristic band of chitosan and A_{1420} is the reference band.

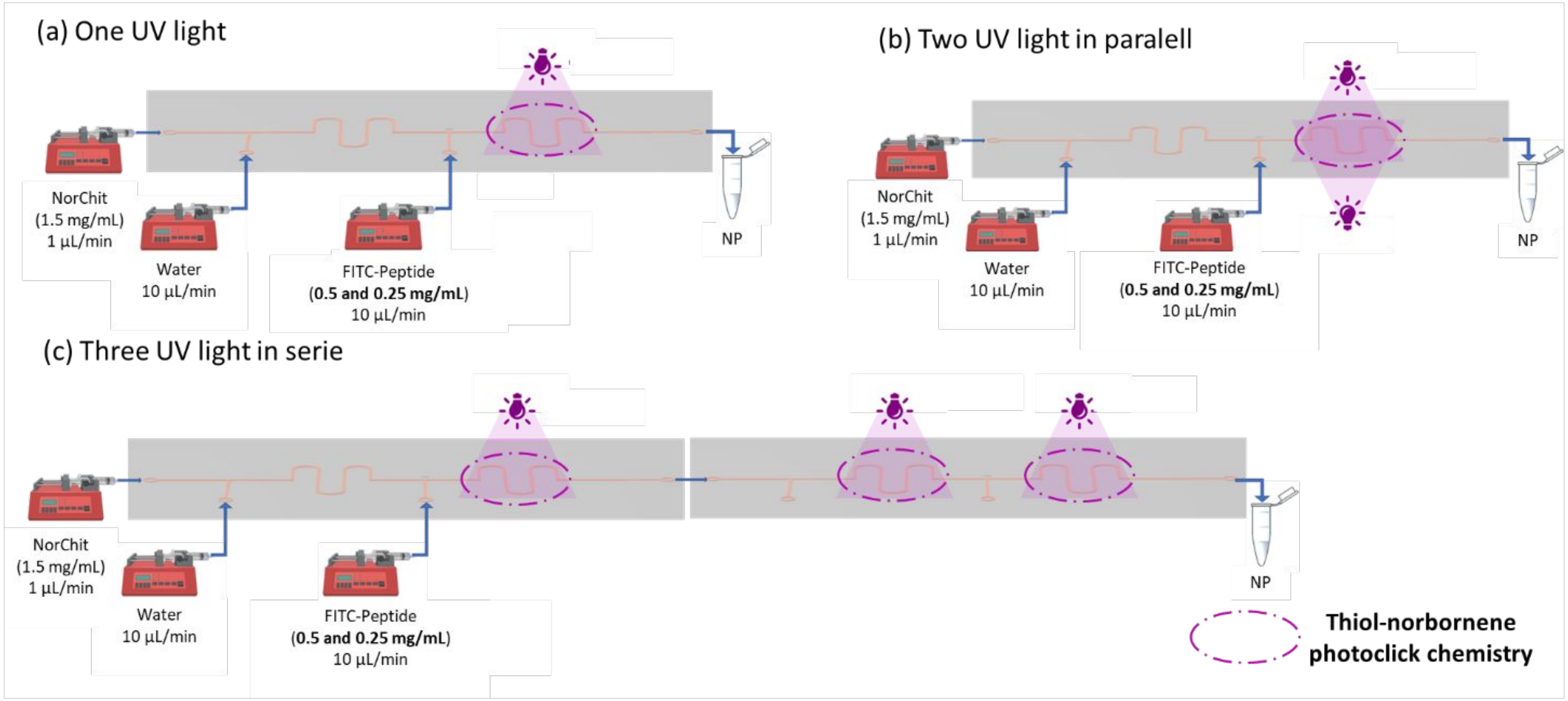

Figure S3. The AMP grafting onto NorChit-NP by thiol–norbornene "photoclick" chemistry (TNPC) was optimized using a fluorescent model peptide (CGGGGRGDSP; with a fluorescein (FITC) tag) in two concentrations (0.50 and 0.25 mg/mL). The conjugation of the peptide onto NorChit-NP was optimized by measuring the grafting yield in three different systems configurations, corresponding to different UV light intensities. The peptide grafting onto NorChit-NP occurred through the incidence of **(a)** only one UV light (intensity of 35 mW/cm^2), **(b)** 2 UV lights in parallel (intensity of above UV light 35 mW/cm^2 and below UV light 75 mW/cm^2), and **(c)** three UV lights in series (35 mW/cm^2 thrice). The distance of superior LED and microfluidic device is 3 cm. The inferior LED in (b) is immediately below the device. The reaction yield was measuring indirectly by measuring the FITC levels (excitation wavelength of 485 nm and emission wavelength of 528 nm, Synergy Mx) after washing the obtained NP.

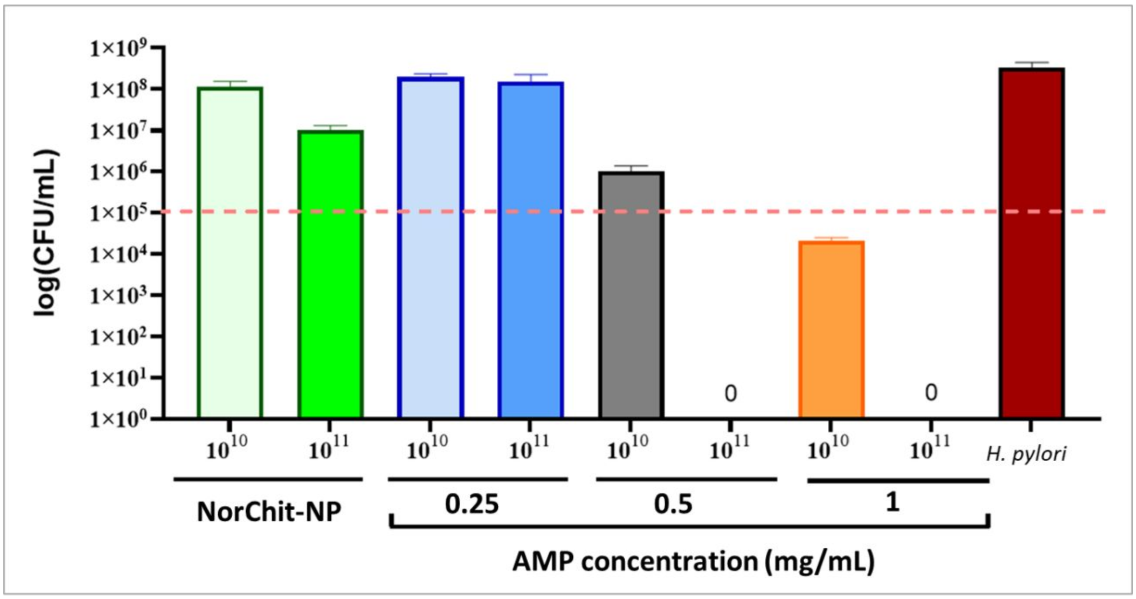

Figure S4. Bactericidal effect of AMP-NP against *H. pylori* J99 strain as gastric bacteria model, in phosphate saline buffer (PBS) for 6 h. Different concentrations of peptide were grafted onto NP: 0.25, 0.50 and 1.00 mg/mL. Results demonstrated that 0.50 and 1 mg/mL in the highest concentration of NP tested (10^{11} NP/mL) had bactericidal effect against this gastric pathogen. Further assays were done using 0.50 mg/mL of AMP, since it was the minimum concentration of peptide needed to have bactericidal effect against *H. pylori*. The dashed line denotes the bactericidal effect (3-log reduction).

Supplementary information references

(1) Alves, P. M.; Pereira, R. F.; Costa, B.; Tassi, N.; Teixeira, C.; Leiro, V.; Monteiro, C.; Gomes, P.; Costa, F.; Martins, M. C. L. Thiol-Norbornene Photoclick Chemistry for Grafting Antimicrobial Peptides onto Chitosan to Create Antibacterial Biomaterials. *ACS Appl Polym Mater* **2022**, *4* (7), 5012–5026. https://doi.org/10.1021/acsapm.2c00563.

(2) Michel, S. E. S.; Dutertre, F.; Denbow, M. L.; Galan, M. C.; Briscoe, W. H. Facile Synthesis of Chitosan-Based Hydrogels and Microgels through Thiol-Ene Photoclick Cross-Linking. *ACS Appl Bio Mater* **2019**, *2* (8), 3257–3268. https://doi.org/10.1021/acsabm.9b00218.

(3) Brugnerotto, J.; Lizardi, J.; Goycoolea, F. M.; Argüelles-Monal, W.; Desbrières, J.; Rinaudo, M. An Infrared Investigation in Relation with Chitin and Chitosan Characterization. *Polymer (Guildf)* **2001**, *42* (8), 3569–3580. https://doi.org/10.1016/S0032-3861(00)00713-8.
